# An explorative study on consequences of abuse on psychological wellbeing and cognitive outcomes in victims of gender-based violence

**DOI:** 10.3389/fpsyg.2024.1367489

**Published:** 2024-07-19

**Authors:** Giulia Lausi, Clarissa Cricenti, Emanuela Mari, Jessica Burrai, Alessandro Quaglieri, Anna Maria Giannini, Benedetta Barchielli

**Affiliations:** ^1^Department of Psychology, Sapienza University of Rome, Rome, Italy; ^2^Department of Dynamic, Clinical Psychology and Health, Sapienza University of Rome, Rome, Italy

**Keywords:** gender-based abuse, emotion regulation, decision making, stress, anxiety, depression, gender-based victimization, cognitive psychology

## Abstract

**Background:**

The issue of gender-based violence has been a public health problem for years. Considering its systemic nature, the possible consequences at the individual level on the psychological and cognitive wellbeing of victims have been examined. The present research aims to explore the differences in the various types and forms of violence.

**Methods:**

A non-probability and convenience sample was used; a total of 83 participants joined the research. Inclusion criteria were minimum age of 18 years, female gender, and knowledge of the Italian language. Two non-parametric One-Way ANOVAs (Kruskal-Wallis) were performed according to the type of violence experienced and the type of self-reported abuse (i.e., no victimization, single victimization, complex victimization).

**Results and discussion:**

Results showed that victims of violence scored higher overall than non-victims on all subscales of the Depression, Anxiety, Stress Scale. Analyses among the three groups-no violence, single violence, and complex violence-showed no differences in any of the dimensions between those who experienced single and complex violence, while differences emerged between the “no victimization” group and the other groups. The results were discussed in relation to the existing literature on the topic, highlighting the limitations and future applications of the collected data.

## Introduction

Gender-based violence (GBV) has long been recognized as a public health issue, which is strictly linked with social structures and necessitating the collective efforts of policymakers and the scientific community. GBV understanding requires the integration of knowledge from diverse disciplines, encompassing social, medical, psychological, and economic sciences. At its core, GBV presents a social and macro-systemic challenge, exerting significant repercussions on the micro-system and individual experiences, fostering a reciprocal influence between these distinct levels. The most prevailing form of violence is Intimate Partner Violence (IPV) which is a pattern of physical, or sexual, or psychological, or emotional abuse directed toward a partner or former partner (Miller and McCaw, 2019; Zara et al., 2020). Extensive research provided important information on the traumatic impact that GBV might have in the personal experience of the victims (Berkowitz, 1993; Tjaden, 2009; Lawrence et al., 2012; Lausi et al., 2021b), although the scientific debate has not yet fully understood and established whether and to what extent different traumas may correspond to different reactions and effects (e.g., the consequences of physical rather than psychological violence, individual rather than collective trauma). The main challenge faced by researchers is the diversity and pervasiveness of traumatic experiences, which they can take the form of minor traumas, or larger traumas involving a serious threat to the person's physical or psychic integrity, sometimes even causing their death (Zara and Gino, 2018). Indeed, it is very often the destructive intensity of such events, both from a physical and psychic point of view, that prevents people from being able to cope effectively, as it is perceived as exceeding available resources (Começanha et al., 2017; Huh et al., 2017; Gambetti et al., 2019). Since the traumatic event involves a painful and unpredictable interruption of the regular flow of events, many people report being unable to lead a satisfying life because of emotions and thoughts related even to events that happened long before Leskin and Sheikh (2002), Johansen et al. (2007), Borges et al. (2021), and Spencer et al. (2023).

### Consequences of GBV on psychological wellbeing

According to World Health Organization data (Krug et al., 2002), IPV may lead to different patterns of sanitary issues, such as HIV infection, sexually transmitted diseases, induced abortion, low birth weight, premature birth, alcohol consumption, suicide, self-harm, and death by homicide. The consequences of domestic violence could therefore have important effects on the health of victims (Lutgendorf, 2019). More generally, victims of GBV seem to be at greater risk of unwanted pregnancies, infections, sexual dysfunctions, and abortion (Pallitto et al., 2013); moreover, in some countries, such as the United States of America, GBV seems to be the primary cause of injury in women (Tjaden and Thoennes, 2001).

Even though most of these findings are based on studies about domestic violence, these consequences are not limited to intra-family violence only: a study conducted in six different European countries (Lukasse et al., 2015) found that among women who reported having an unwanted pregnancy, 24.5% were abused during their lifetime and 38.5% were recently abused. The consequences of GBV are far-reaching, extending beyond immediate health concerns to impact the long-term wellbeing and decision-making capacity of victims. Such experiences have been observed to manifest in various psychological reactions, including depression, anxiety, and a diminished sense of self-esteem.

The health consequences of abuse may also have long-term secondary outcomes which could be related to the primary ones. Individuals suffering from depression may experience impairments in the executive (Snyder, 2013) and cognitive functions, e.g., attention and memory. Particularly, a recent study (Lawlor et al., 2020) found that individuals suffering from Major Depressive Disorder seem to need more time to make decisions and show biased decision-making strategies (Adolphs et al., 1996; Bechara et al., 1998). Moreover, some relationships between the physiological response to stress and functions such as attention, executive functions, decision-making have been highlighted (Lebois et al., 2016). Symptoms' severity seems to be related to the individual tendency to suppress painful contents and mitigate planification strategies (Huys et al., 2012; Montague et al., 2012); deciding implies a simultaneous evaluation of present stimuli and possible choices (Aupperle et al., 2012; Palmiero et al., 2020; Gambetti et al., 2022) which subconsciously involves the information reduction while reasoning (Kahneman and Frederick, 2002; Shah and Oppenheimer, 2008; Evans and Stanovich, 2013), also affected by moods and emotions in the post-reasoning evaluations and therefore decision-making processes (Bower, 1981; Forgas, 1989; Schwarz and Clore, 1996; Yildiz and Eldeleklioglu, 2021). While considerable research has explored the physical and mental health consequences of GBV, there remains a paucity of studies specifically investigating the effects of violence on the decision-making processes of victims (Wiebe and Janssen, 2001; Leung et al., 2002; Pallitto et al., 2005; Bourassa and Bérubé, 2007; Ely and Otis, 2011; Roth et al., 2011; Lausi et al., 2023). The complex nature of decision-making within the context of GBV presents challenges in defining a cohesive framework for examination, as outcomes may vary considerably based on diverse factors, including the victim's perception of violence, control dynamics, and emotional attitudes. To advance our understanding, it is crucial to explore the interplay between GBV and decision-making processes comprehensively.

### Aims

The present research seeks to obtain data which will help to address the research gap within experiencing GBV and the long-term effects of trauma on cognitive functions. More specifically, this paper aims to explore differences in emotional regulation, decision-making strategies, direct and indirect strategies of psychological abuse and anxiety, stress and depression between people who have experienced different types of violence and between people who have experienced different forms of abuse (Daigle et al., 2008; Walsh et al., 2020; Daigle and Hawk, 2022) and in assessing decision-making and risk-taking through the use of an implicit measure, in a sample of victims of abuse recruited from anti-violence centers and a control group. Since to the best of our knowledge, no previous studies investigated the differences among the different forms of abuse in cognitive decision-making and risk-taking, the analyses concerning the cognitive task are explorative only.

In particular, the following assumptions were made:

**H1:** There is a relationship between implicit measurements (cognitive task) and stress, anxiety, and depression; we expect that people with higher scores in the DASS will show a longer response time (Lebois et al., 2016; Lawlor et al., 2020).

**H2:** The implicit measurement of decision-making (measured by the Millisecond Gambling Task) may be predicted by the explicit variables (measured by the questionnaire) with differences among the no-violence, single-violence, and complex violence victims.

## Materials and methods

### Participants

A non-probabilistic and convenience sampling was used, and a total of 83 participants joined the research. Inclusion criteria were being at least 18 years old, being a female and being Italian speaker (Table 1).

**Table 1:** Descriptive statistics.

	***N***	**M**	**SD**
**Age**	83	24.3	8.57
			***N*** **(%)**	
Marital status	Unmarried, in a relationship	49 (59.0%)	
	Single	27 (32.5%)	
	Married	5 (6.0%)	
	Legal Separation	1 (1.2%)	
	Divorced	1 (1.2%)	
Experienced psychological violence	Yes	47 (43.4%)	
	No	36 (56.6%)	
Experienced economic violence	Yes	4 (4.8%)	
	No	79 (95.2%)	
Experienced sexual violence	Yes	21 (25.3%)	
	No	62 (74.7%)	
Experienced physical violence	Yes	9 (10.8%)	
	No	74 (89.2%)	
Did not experience violence		27 (32.5%)	
Kind of violence	No violence	27 (33.3%)	
	Single violence	32 (39.5%)	
	Complex violence	22 (27.2%)	

M, Mean; SD, Standard deviation.

### Procedure

Data have been collected throughout Italy using the Qualtrics Platform for online surveys. The questionnaire was launched in January 2022 and data were collected until April 2022. All study procedures were carried out according to Helsinki Declaration and approved by the Institutional Review Board of the Department of Psychology, the University of Rome “Sapienza” with protocol number 0001446 (5/07/2021).

The present study employed a quantitative approach, through an online questionnaire, which was used to collect the target information, and a cognitive task used to collect implicit measures. The questionnaire was administered through Qualtrics platform; at the end of the questionnaire a short link redirected participants to the Inquisit web platform to perform the Millisecond gambling task. Both questionnaire and task were administered through computers and laptops.

The online survey was spread through various channels, such as working platforms, word of mouth and social networks, to reach as many participants as possible from the general population. Participants were recruited through a snowball sampling method: the participants from the researchers' networks referred to other people who accepted participating to the study.

The victims of violence (*N* = 56) were recruited through shelters and in addition, we made sure to contact women's anti-violence and victim support centers to promote the research and propose the participation of victims on a voluntary basis. This was done to ensure that the voices of those affected by violence were heard and to recognize and value the contribution of victims to the research. Furthermore, special efforts were made to ensure that the survey was accessible so that victims could participate without fear of retribution or discrimination. Two victims did not declare the kind of violence episodes (single or multiple violence); therefore, they were not considered for the final analysis, leading to a total number of 54 victims.

### Questionnaire

#### Demographic information

For demographic information of the sample data on age, marital status, whether they had experienced violence and what type of violence they had experienced were collected.

#### Depression Anxiety Stress Scale

The Depression Anxiety Stress Scale is the shortened form of the DASS-42 (Lovibond and Lovibond, 1995), a scale used to measure depression, anxiety, and stress (Scholten et al., 2017). It consists of 21 items that participants must answer according to a 4-point Likert scale, indicating how often the situation described has occurred in the last 7 days. The Stress subscale showed a Cronbach's alpha of 0.862, the Anxiety subscale showed a Cronbach's alpha of 0.811, while Depression showed a value of 0.897.

#### Difficulties in Emotion Regulation Scale-20

The Difficulties in Emotion Regulation Scale (DERS) is a self-report measure developed by Gratz and Roemer (2004) to assess difficulties in emotion regulation. In the present study, the reduced version DERS- 20 was used (Lausi et al., 2020), which consists of five factors: lack of acceptance (Non acceptance, α = 0.912) of one's own distress; difficulties in distraction (Goals, α = 0.891), which reflects the difficulty in concentrating and finishing a task when upset; lack of control (Impulse, α = 0.906) in the face of negative emotions; difficulties in recognizing (Clarity, α = 0.822) one's own emotions and self-awareness (Awareness, α = 0.832). The latter scale measures the tendency to pay attention to one's emotions. The items are rated on a five-point Likert scale ranging from 1 (almost never) to 5 (almost always).

#### Scale of Psychological Abuse in Intimate Partner Violence

The Scale of Psychological Abuse in Intimate Partner Violence (EAPA-P) (Lausi et al., 2021a) consists of 19 items that indicate direct and indirect strategies of psychological abuse between partners. Specifically, direct strategies include emotional abuse, imposition of one's own thoughts and imposition of a submissive role (α = 0.868). Among indirect strategies (α = 0.910), on the other hand, the scale identifies isolation, control and manipulation of information, and control of personal life.

#### Melbourne Decision-Making Questionnaire

The Melbourne Decision-Making Questionnaire (MDMQ) is an instrument developed by Mann et al. (1997) that aims to investigate the individual's approach to decision-making. Four sub-scales can be identified from its 22 items: vigilance (V, α = 0.758), hypervigilance (H, α = 0.809), procrastination (P, α = 0.759) and avoidance (A, α = 0.917). The latter attitude consists of avoiding taking responsibility and leaving decisions to be made by others.

The items are rated on a three-point scale ranging from “it is not true for me” to “it is true” (Filipe et al., 2020).

### Millisecond gambling task

The Millisecond Gambling Task is a task developed to assess decision-making and risk-taking, adapted from the Cambridge Gambling Task (Rogers et al., 1999).

Participants are given 10 boxes which are red and blue on a different ratio (i.e., 1:9; 2:8; 3:7; 4:6) and told that a yellow token is hidden under one of these boxes. Once they decide on the color, participants are asked to bet points on their choice. The bets are either in ascending or descending order. If they win, the bet number is added to the total points, if they lose the number is taken away. The MGT can measure several aspects: risk-taking, represented by the betting ratio where participants choose the optimal color; the quality of the decision-making process, represented by the percentage of trials in which the participant chooses the color with the most boxes; the impulsiveness/aversion to delay, revealed by the choice of the percentages that are shown first deliberation time, given by the time between the start of the trial and the choice of the percentage; and risk adjustment, which captures the tendency of participants to bet more when the odds are in their favor (Zois et al., 2014; Yazdi et al., 2019; Romeu et al., 2020).

### Data analysis

Statistical analyses were conducted using SPSS (Statistical Package for Social Science; version 27.0; IMB SPSS; Armonk, NY, USA). Descriptive analyses of sample characteristics were performed; then, the assumption for normality was checked for all the scales. Since the normality assumption was violated, a non-parametric One-Way ANOVA (Kruskal-Wallis) was performed among the three groups according to the self-reported type of the experienced form of abuse (i.e., no victimization, single victimization, complex victimization) (Daigle et al., 2008; Walsh et al., 2020; Daigle and Hawk, 2022). Statistical significance in the *post-hoc* analysis was determined using Dwass-Steel-Critchlow-Fligner (DSCF) pairwise comparisons and defined as *p* < 0.05. A Spearman non-parametric correlation was performed among the variables measured through the DASS subscales and the Millisecond Gambling Task outcomes. A linear regression analysis was performed among the three groups with Millisecond Gambling Task total point as dependent variable. The idea of including questionnaire scales as predictors is given by the possible variables that generally influence decision-making and risk-taking.

## Results

### Differences among forms of violence in depression, anxiety, and stress

A non-parametric One-Way ANOVA was performed to reveal any statistically significant differences in Depression, Anxiety and Stress among victims of single and complex violence and non-victims. Results revealed no statistically significant differences in any of the subscales; the Dwass-Steel-Critchlow-Fligner (DSCF) pairwise comparisons showed that women who did not experience violence showed statistically lower scores in Stress (M = 10.9; SD = 7.26) than women who experienced single violence (M = 15.0; SD = 8.89) (Table 2).

**Table 2:** Non-parametric One-Way ANOVA in depression, anxiety, stress.

		**X^2^**	**df**	**p**	**η^2^**	**W**	***p***
Stress	noV vs. singleV	5.734	2	0.057	-	3.40	0.043^*^
	noV vs. compV					1.74	0.435
	singleV vs. compV					−1.32	0.618
Anxiety	noV vs. singleV	4.562	2	0.102	-	2.560	0.166
	noV vs. compV					−0.471	0.941
	singleV vs. compV					−2.511	0.573
Depression	noV vs. singleV	5.631	2	0.060	-	2.731	0.130
	noV vs. compV					3.081	0.075
	singleV vs. compV					0.324	0.972

^*^p <0.05; ^**^p <0.01; ^***^p <0.001. noV, no violence; singleV, single violence; compV, complex violence.

### Differences among forms of violence in emotion dysregulation

A non-parametric One-Way ANOVA was then performed to reveal any statistically significant differences in Difficulties in Emotion Regulation subscales among victims of single and complex violence and non-victims. Results revealed significant differences in the non-Acceptance subscale. Through Dwass-Steel-Critchlow-Fligner (DSCF) pairwise comparisons differences among the three groups were investigated. Women who did not experience violence showed statistically lower scores (M = 9.04; SD = 4.31) than women who experienced both single violence (M = 12.3; SD = 5.77) and complex violence (M = 13.1; SD = 5.85) (Table 3).

**Table 3:** Non-parametric One-Way ANOVA in emotion disregulation.

		**X^2^**	**df**	**p**	**η^2^**	**W**	***p***
Awareness	noV vs. singleV	3.351	2	0.187	-	1.72	0.443
	noV vs. compV					−1.23	0.659
	singleV vs. compV					−2.34	0.222
Non-acceptance	noV vs. singleV	8.027	2	0.018^*^	0.042	3.380	0.044^*^
	noV vs. compV					3.505	0.035^*^
	singleV vs. compV					0.638	0.894
Goals	noV vs. singleV	4.755	2	0.093	-	2.21	0.261
	noV vs. compV					2.90	0.100
	singleV vs. compV					1.10	0.716
Impulse	noV vs. singleV	4.395	2	0.111	-	2.895	0.101
	noV vs. compV					1.937	0.357
	singleV vs. compV					−0.775	0.848
Clarity	noV vs. singleV	0.673	2	0.071	-	−0.545	0.922
	noV vs. compV					−1.147	0.696
	singleV vs. compV					−0.694	0.876

^*^p <0.05; ^**^p <0.01; ^***^p <0.001. noV, no violence; singleV, single violence; compV, complex violence.

### Differences among forms of violence in strategies of psychological violence

A non-parametric One-Way ANOVA was performed to investigate differences between victims of single and complex violence and non-victims in the EAPA-P scale. Results showed no statistically significant differences in both Direct and Indirect strategies of psychological violence. The Dwass-Steel-Critchlow-Fligner (DSCF) pairwise comparisons showed differences among non-victims (M = 8.93; SD = 2.60) and complex violence victims (M = 11.3; SD = 5.15) in Direct strategies (Table 4).

**Table 4:** Non-parametric One-Way ANOVA in strategies of psychological violence.

		**X^2^**	**df**	**p**	**η^2^**	**W**	***p***
Direct Strategies	noV vs. singleV	4.912	2	0.086	-	1.62	0.485
	noV vs. compV					3.31	0.050^*^
	singleV vs. compV					1.42	0.576
Indirect Strategies	noV vs. singleV	1.259	2	0.533	-	0.893	0.803
	noV vs. compV					1.518	0.531
	singleV vs. compV					0.921	0.792

^*^p <0.05; ^**^p <0.01; ^***^p <0.001. noV, no violence; singleV, single violence; compV, complex violence.

### Differences among forms of violence in decision-making strategies

A non-parametric One-Way ANOVA was performed to investigate differences among victims of single and complex violence and non-victims in the Melbourne Decision Making Questionnaire, showing no statistically significant differences in any of the subscales nor in the comparison among groups (Table 5).

**Table 5:** Non-parametric One-Way ANOVA in decision making strategies.

		**X^2^**	**df**	**p**	**η^2^**	**W**	***p***
Procrastination	noV vs. singleV	3.688	2	0.158	-	2.708	0.135
	noV vs. compV					1.645	0.475
	singleV vs. compV					−0.740	0.860
Hypervigilance	noV vs. singleV	2.057	2	0.358	-	−1.333	0.614
	noV vs. compV					−1.975	0.343
	singleV vs. compV					−0.818	0.832
Vigilance	noV vs. singleV	0.697	2	0.706	-	−0.378	0.961
	noV vs. compV					−1.043	0.741
	singleV vs. compV					1.001	0.759
Avoidance	noV vs. singleV	5.080	2	0.079	-	1.31	0.622
	noV vs. compV					3.18	0.063
	singleV vs. compV					2.02	0.327

^*^p <0.05; ^**^p <0.01; ^***^p <0.001. noV, no violence; singleV, single violence; compV, complex violence.

### Correlations among DASS and millisecond gambling task

According to the hypotheses, the Millisecond Gambling Task (MGT) scores were correlated with the subscales of the DASS. Positive correlations were found in the “meanrt_deliberation” scores, i.e., all those scores that measure the average reaction time taken to decide (where the numbers represent the ratio of the boxes), in both the stress and depression subscales (Table 6).

**Table 6:** Correlations among DASS and MGT.

	**Anxiety**	**Stress**	**Depression**
totalpoints	0.048	−0.070	−0.100
meanrt_deliberation	0.047	0.337^**^	0.249^*^
meanrt_deliberation46	0.046	0.279^*^	0.195
meanrt_deliberation37	0.065	0.224^*^	0.219^*^
meanrt_deliberation28	0.013	0.302^**^	0.240^*^
meanrt_deliberation19	0.063	0.288^**^	0.207
percent_bestchoice	−0.030	−0.197	−0.205
meanrt_betlatency_A	0.026	0.024	−0.034
meanrt_betlatency_D	−0.146	−0.015	0.131
mean_percentbet_total	0.088	−0.023	−0.145
mean_percentbet_A	0.032	0.022	−0.034
mean_percentbet_D	0.168	0.027	−0.131
mean_percentbet46	0.117	0.181	0.033
mean_percentbet37	0.092	−0.003	−0.121
mean_percentbet28	0.088	−0.053	−0.155
mean_percentbet19	0.070	−0.127	−0.163

^*^p <0.05; ^**^p <0.01; ^***^p <0.001. meanrt_deliberation: average reaction time taken to decide; percent_bestchoice: percentage of trials where a participant chooses the color with more boxes; meanrt_betlatency (A or D): time needed to decide when placing a bet (A for Ascending Bet, D for Descending Bet); mean_percentbet: the average percentage of money that participants choose to bet.

### Regression model for decision-making

Three multiple logistic regression analyses were performed with the “totalpoint” of Millisecond Gambling Task as dependent variable in the three groups: no violence, single violence, and complex violence victims. The variables inserted as predictors were all the subscales of DASS, DERS-20, EAPA-P, and MDMQ.

#### “No violence” group

The first multiple logistic regression was conducted on the “no violence” group; the results showed that the model in this group the total point of Millisecond Gambling Task was not statistically significant [F_(14, 12)_ = 1.35, *p* = 0.304, R^2^ = 0.612] and so were the predictors in the model (Tables 7, 8).

**Table 7:** “No violence” regression.

			**Overall model test**			

**Model**	**R**	**R** ^2^	**F**	**df1**	**df2**	***p***
1	0.782	0.612	1.35	14	12	0.304

Dependent variable “totalpoint”.

**Table 8:** Predictors of the “No violence” regression model.

**Predictor**	**Estimate**	**SE**	**t**	***p***
Intercept	18,513.5	8,749	2.116	0.056
Stress	−598.7	306	−1.954	0.074
Anxiety	54.2	207	0.262	0.798
Depression	−56.6	213	−0.265	0.795
Awareness	−494.0	504	−0.980	0.346
Clarity	557.0	456	1.222	0.245
Non-acceptance	133.1	401	0.332	0.746
Goals	−449.4	331	−1.357	0.200
Impulse	556.1	446	1.247	0.236
Direct Strategies	1,104.6	686	1.610	0.133
Indirect Strategies	−2,073.4	976	−2.124	0.055
Procrastination	611.6	793	0.771	0.456
Vigilance	−153.3	422	−0.364	0.723
Avoidance	−593.1	430	−1.381	0.193
Hypervigilance	−86.9	605	−0.143	0.888

Dependent variable “totalpoint”.

#### “Single violence” group

The second multiple logistic regression was conducted on the “single violence” group; the results showed that the model in this group the total point of Millisecond Gambling Task statistically significant and explained about 70% of the variance [F_(14, 16)_ = 2.67, *p* = 0.031, R^2^ = 0.700]; according to the model, Stress, Goals and Impulse subscales are the variables that significantly predict the model (Tables 9, 10).

**Table 9:** “Single violence” regression model.

			**Overall model test**			

**Model**	**R**	**R** ^2^	**F**	**df1**	**df2**	**p**
1	0.837	0.700	2.67	14	16	0.031

Dependent variable “totalpoint”

**Table 10:** Predictors of the “Single violence” regression model.

**Predictor**	**Estimate**	**SE**	**t**	***p***
Intercept	9,228.1	6,063	1.5219	0.148
Stress	−529.7	231	−2.2889	0.036^*^
Anxiety	574.5	185	3.0984	0.007^**^
Depression	−83.9	131	−0.6420	0.530
Awareness	−294.6	235	−1.2549	0.228
Clarity	155.1	415	0.3738	0.713
Non-acceptance	23.8	231	0.1029	0.919
Goals	536.5	250	2.1487	0.047
Impulse	−464.5	220	−2.1155	0.050^*^
Direct Strategies	39.7	278	0.1430	0.888
Indirect Strategies	783.3	680	1.1520	0.266
Procrastination	114.5	530	0.2161	0.832
Vigilance	−482.5	411	−1.1754	0.257
Avoidance	−426.3	310	−1.3741	0.188
Hypervigilance	19.7	408	0.0483	0.962

Dependent variable “totalpoint”. ^*^p < 0.05; ^**^p < 0.01.

#### “Complex violence” group

The last multiple logistic regression was conducted on the “complex violence” group; the results showed that the model in this group the total point of Millisecond Gambling Task was not statistically significant (F _(14, 5)_ =0.739, p =0.700, R^2^ =0.674) and so were the predictors in the model (Tables 11, 12).

**Table 11:** “Complex violence” regression model.

			**Overall model test**			

**Model**	**R**	**R** ^2^	**F**	**df1**	**df2**	**p**
1	0.821	0.674	0.739	14	5	0.700

Dependent variable “totalpoint”.

**Table 12:** Predictors of the “Complex violence” regression model.

**Predictor**	**Estimate**	**SE**	**t**	***p***
Intercept	7,247.7	12,858	0.5637	0.597
Stress	29.7	348	0.0853	0.935
Anxiety	199.8	336	0.5945	0.578
Depression	263.7	421	0.6259	0.559
Awareness	−90.6	561	−0.1615	0.878
Clarity	−1,705.1	1,020	−1.6715	0.155
Non-acceptance	−778.0	827	−0.9403	0.390
Goals	461.3	538	0.8574	0.430
Impulse	724.0	1,139	0.6357	0.553
Direct Strategies	1,559.7	1,360	1.1467	0.303
Indirect Strategies	−2,501.8	2,153	−1.1620	0.298
Procrastination	−578.3	961	−0.6015	0.574
Vigilance	−414.7	953	−0.4354	0.681
Avoidance	−331.9	992	−0.3347	0.751
Hypervigilance	1,245.3	1,644	0.7577	0.483

Dependent variable “totalpoint”.

## Discussion

In general, no differences emerge among groups for most of the considered variables. However, the comparison among the three groups revealed statistically significant differences within the Stress subscale of the DASS, the Non-Acceptance subscale of the DERS-20, and the Direct Strategies subscale of the EAPA-P. Specifically, it was found that the group that had not experienced violence showed lower scores in the Stress subscale compared to the group that had experienced a single form of violence. This finding can be explained by the impact of trauma on violence victims, irrespective of the number of violent experiences they may have encountered (Daigle et al., 2008; Walsh et al., 2020; Daigle and Hawk, 2022; Petersen et al., 2022). If we solely observe the means, disregarding significance, it becomes apparent that individuals who have experienced a single form of violence exhibit higher average scores compared to all other groups. Several studies (Green et al., 2000; Hossain et al., 2010; Dworkin et al., 2017) highlighted that traumatic and stressful events can have a greater impact when tied to a single incident rather than repetitive trauma. It would be interesting to investigate whether this phenomenon also occurs in violence victims, leading to the hypothesis that the lack of statistically significant differences among groups merely represents a pattern already studied in other traumatic experiences. Women who claim to have never experienced any form of violence showed lower levels of emotions non-acceptance than who reported experiencing a single violence event and women who reported experiencing complex violence. With an increase in stressful or traumatic events, the difficulty in accepting one's emotions also intensifies (Paivio and Laurent, 2001; Follette et al., 2006). This data could be further explored by focusing specifically on emotional regulation and emotions related to episodes of abuse. However, due to the limited sample size in this study, further hypotheses cannot be advanced. Finally, a statistically significant difference emerged within the Direct Strategies of Psychological Violence subscale between those who reported never experiencing violence and those who have experienced complex violence. We may presume that this data can be interpreted in reverse, i.e., there are no differences between those who claimed to have never experienced violence and those who experienced a single form of violence. This is because psychological violence is often concealed and subtle, and victims may not have recognized or reported it (Marshall, 1999; Follingstad, 2007; McHugh et al., 2013; Samios et al., 2020). Similarly, there may be no differences between those who experienced a single form of violence and those who experienced complex violence since psychological violence is often a precursor to other forms of violence and may be present in both groups.

To test the hypotheses of a relationship between implicit measurements (cognitive task) and stress, anxiety, and depression (**H1**) regression analysis was conducted. According to previous research (Lebois et al., 2016; Lawlor et al., 2020) it was hypothesized that people with higher scores in the DASS would show longer response time and lower risk-taking. Results showed significant results in the response time, according to the hypotheses. It's also worth noting that even variables with no significant correlation still go in the expected direction.

To test **H2** the total score of the Millisecond Gambling Task was set as the dependent variable in a linear regression model, with the questionnaire variables set as predictors, for each group. The predictive model in the group of women who did not experience violence showed a good explained-variance value, although the predictors were not significant. The same result also emerged in the complex violence group. In contrast, the pattern in the single violence group was significant, and the variables Stress, Anxiety, Goals, and Impulse were significant within the model. However, the sample size is small, so no inferences can be made either on the validity of the model or on individual predictors.

### Limits

Despite the considerations made so far, the research has some limitations that it is important to highlight. First, the sample size should be expanded to be able to make statistical inferences that can be discussed more reliably, even though victims come from shelters making the answers more accountable than a more shared online questionnaire. Replicating the research by collaborating with more shelters could allow the sample to be enlarged enough to consider conducting a mediation analysis to better understand the relationships between the factors and to include decision-making style as a possible variable in addition to decision-making strategies. Moreover, following the considerations that emerged from the study, one could include a specific screening for possible disorders (e.g., PTSD, substance abuse...) that may be closely linked to the variables examined, especially referred to Depression, Anxiety, Stress Scale symptoms. Finally, there is no detail on time differences between the forms of violence: this information, although important, was not included following the specific suggestion of the anti-violence centers that helped with data collection, as it is part of the sensitive information that is asked of the victims.

### Future perspectives

The limits of the present research allow us to identify which may be the future perspectives of the study and, more specifically, of cognitive research in victims of violence. First, the sample of the present study should be enlarged to carry out a more accurate investigation of the implicit variables examined. In addition, longitudinal studies should be carried out, starting from the present research, to allow a broader interpretation of the data, including the addition of a semi-structured interview, so that the individual experience of the victim is not lost when interpreting the quantitative data measuring common experience. In addition, the use of qualiquantitative data in an area that is still under-investigated within abusive contexts (i.e., cognitive processes) can be useful in the construction of training paths for those professionals working directly with victims but also in the development of evidence-based clinical practices that consider not only the short-term effects of gender-based violence but also the long-term effects.

### Diversity assessment

Diversity factors can significantly impact the experiences of GBV victims. While the present sample primarily consisted of Italian-speaking females, it is important to acknowledge that the effects of GBV may vary across diverse cultural, linguistic, and socio-economic backgrounds. Future research should prioritize a more diverse participant pool to capture the experiences and consequences of GBV among various populations. Additionally, investigating the intersectionality of diversity factors, such as race, ethnicity, disabilities, and socio-economic status, is essential for understanding how these overlapping identities influence the overall wellbeing among victims.

### Conclusion

This study set out to explore the influence of different forms of GBV experience on psychological wellbeing and cognitive outcomes in a sample of victims and non-victims. The data collected, although mainly with an exploratory purpose, allow several considerations to be made on the topic of gender-based violence. First, there are differences between those who have and have not experienced violence in their lifetime. These differences, however, do not follow specific patterns. It emerged that there are differences between those who have not experienced violence and those who have—both single and complex—but that there are no differences between groups of people who have experienced one or more forms of violence. Taken together, these results suggest that once a victim has experienced a traumatic event, such as one of the forms of abuse under investigation, they react not only in the immediate term but may develop coping strategies and forms of resilience that they will also use in the long term (McCann and Pearlman, 1990; Berkowitz, 1993; Gutner et al., 2006; Tjaden, 2009; Lawrence et al., 2012; Lausi et al., 2021b). However, these results must be approached with come caution to avoid a risk of “normalization” of the violence within an individual's life in the long term, a risk that could lead to a devaluation of the pain or even in extreme cases to the attribution of responsibility on the victim of complex violence (Humphrey, 2003; McCarry and Lombard, 2016).

Another aspect to be taken into consideration concerns, more generally, the interpretation of data. The risk, when we talk about gender-based violence and decision-making or risk-taking, is once again to find ourselves saying that the victim is such because she does not use the correct decision-making strategies or because she is “reckless”: studies in victims of violence, for obvious ethical reasons, cannot predict “pre” violence, so we cannot know the connection between the decision-making strategies before the traumatic event and the event itself. What we do know is that numerous studies have shown how, in situations of similar or even lower stress, coping strategies and decision-making and risk-taking patterns changed (Adolphs et al., 1996; Bechara et al., 1998; Huys et al., 2012; Montague et al., 2012; Lebois et al., 2016; Taylor et al., 2018; Minooee et al., 2020). And it is only in the “post-violence” phase that we are interested in knowing how victims behave, and what the cognitive effects of violence may be, not to judge but to be able to implement support programmes for victims that are effective and aimed at acquiring new skills in risk assessment, decision-making and emotional regulation.

## Data availability statement

The raw data supporting the conclusions of this article will be made available by the authors, without undue reservation.

## Ethics statement

The studies involving humans were approved by Institutional Review Board of the Department of Psychology, the University of Rome “Sapienza” with protocol number 0001446 (5/07/2021). The studies were conducted in accordance with the local legislation and institutional requirements. The participants provided their written informed consent to participate in this study.

## Author contributions

GL: Conceptualization, Data curation, Formal analysis, Investigation, Methodology, Project administration, Resources, Software, Supervision, Validation, Visualization, Writing – original draft, Writing – review & editing. CC: Conceptualization, Methodology, Writing – original draft. EM: Writing – review & editing. JB: Writing – review & editing. AQ: Writing – review & editing. AG: Supervision, Validation, Writing – review & editing. BB: Writing – original draft.
